# Draft Genome Sequence of Lactobacillus plantarum CRL681, Isolated from Argentinean Artisanal Fermented Sausages

**DOI:** 10.1128/MRA.01629-18

**Published:** 2019-04-11

**Authors:** Silvina Fadda, Julio Villena, Leonardo Albarracin, Lucila Saavedra, M. Aminul Islam, Graciela M. Vignolo, Haruki Kitazawa, Elvira Maria Hebert

**Affiliations:** a Reference Centre for Lactobacilli (CERELA-CONICET), Tucuman, Argentina; b Food and Feed Immunology Group, Laboratory of Animal Products Chemistry, Graduate School of Agricultural Science, Tohoku University, Sendai, Japan; c Scientific Computing Laboratory, Computer Science Department, Faculty of Exact Sciences and Technology, National University of Tucuman, Tucuman, Argentina; d Livestock Immunology Unit, International Education and Research Centre for Food Agricultural Immunology (CFAI), Graduate School of Agricultural Science, Tohoku University, Sendai, Japan; Indiana University, Bloomington

## Abstract

Lactobacillus plantarum CRL681 was isolated from Argentinean artisanal fermented sausages. Here, the draft genome sequence of the CRL681 strain is described. The reads were assembled into contigs with a total estimated size of 3,370,224 bp. A total of 3,300 open reading frames (ORFs) were predicted, including 3,126 protein-coding sequences. The draft genome sequence of *L. plantarum* CRL681 will be useful for understanding the organism’s metabolic activities and for biotechnological applications.

## Abstract (precis)

Lactobacillus plantarum CRL681 was isolated from Argentinean artisanal fermented sausages. Here, the draft genome sequence of the CRL681 strain is described.

## ANNOUNCEMENT

Lactobacillus plantarum CRL681, a strain originally isolated from an artisanal Argentinean fermented sausage, has an efficient acidogenic activity that guarantees safety and texture development during ripening of fermented sausages (1–7). The CRL681 strain possesses different aminopeptidases, and it has been demonstrated to have the ability to contribute to meat protein degradation by promoting the activity of muscle proteolytic enzymes (2, 3, 5, 8). Detailed peptidomic studies confirmed its peptidogenic ability and its capacity to increase free amino acid contents when inoculated into raw meat or fermented-meat models (6, 8, 9). On the other hand, *L. plantarum* CRL681 is capable of degrading biogenic amines *in vitro* and lacks the ability to produce them from amino acids, indicating the absence of amino oxidase and amino decarboxylase activities, respectively (4). In addition, the CRL681 strain has remarkable bioprotective potential due to the high inhibitory activity toward Escherichia coli O157:H7 (10).

*L. plantarum* CRL681 was grown for 12 h at 30°C (final log phase) in Man-Rogosa-Sharpe (MRS) agar (Oxoid, Cambridge, UK). A single colony was picked for DNA isolation. Library preparation was performed using a Nextera XT DNA library prep kit following the manufacturer’s protocol. Briefly, 1 ng of DNA (5 μl of the sample normalized to 0.2 ng/μl) was submitted to enzymatic fragmentation by transposons and end labeling, followed by adapter ligation, amplification, and purification of DNA fragments. The *L. plantarum* CRL681 genomic DNA was sequenced with the 2 × 150-bp paired-end read length sequencing protocol of the Illumina MiSeq platform. The quality of the reads was controlled using FastQC (11), and the generated sequencing reads were filtered to remove low-quality reads using Prinseq (12) with the following parameters: Min_len, 150; Trim_left, 15; Trim_right, 10; and Min_qual_mean, 25. SPAdes v3.11.1 (13) was used for *de novo* assembly with an *N*_50_ value of 449,362 bp. The sequencing protocol generated 191× mean coverage of the genome. The CRL681 draft genome sequence contains 28 contigs with an average GC content of 44.3% and a total estimated size of 3,370,224 bp.

The Rapid Annotations using Subsystems Technology (RAST) server was used for functional annotation of predicted genes (14). A total of 3,300 open reading frames (ORFs) were predicted, including 3,126 protein-coding sequences, 61 tRNAs, 17 rRNAs, and 4 noncoding RNAs (ncRNAs). No clustered regularly interspaced short palindromic repeats (CRISPRs) were found in the genome by using CRISPRFinder (15). Default parameters were used in all of the bioinformatic analyses.

Fifteen putative peptidases were detected when the genome of *L. plantarum* CRL681 was analyzed by RAST (12) and submitted to the online BLAST search tool on the MEROPS peptidase database (16), demonstrating the peptidolytic potential of this strain. Additionally, a choloylglycine hydrolase gene for bile hydrolysis was found in *L. plantarum* CRL681 that could be involved in its ability to survive in the gastrointestinal tract (17). Clusters of genes involved in the biosynthesis of folate and riboflavin were also found in the CRL681 genome.

The draft genome sequence of *L. plantarum* CRL681 will be useful for understanding its metabolic activities and biotechnological applications.

### Data availability.

This whole-genome shotgun project has been deposited at DDBJ/EMBL/GenBank under the accession number QOSF00000000. The version described here is QOSF01000000. The SRA/DRA/ERA accession number is ERP111695. The BioSample and BioProject numbers are SAMN09649875 and PRJNA480792, respectively.
